# Promising results of a resource- and activity-oriented intervention integrating rehabilitation into palliative care in people with advanced cancer: A feasibility study testing outcome measures

**DOI:** 10.1017/S1478951524001652

**Published:** 2025-02-07

**Authors:** Marie Brunsgaard Laursen, Marc Sampedro Pilegaard, Karen la Cour

**Affiliations:** 1 User Perspectives and Community-based Interventions, Research Group for Occupational Science, Department of Public Health, University of Southern Denmark, Odense M, Denmark; 2 DEFACTUM, Central Denmark Region, Aarhus, Denmark; 3 Department of Social Medicine and Rehabilitation, Gødstrup Hospital, Herning, Denmark; 4 Department of Clinical Medicine, Aarhus University, Aarhus, Denmark

**Keywords:** palliative care, rehabilitation, quality of life, neoplasms, feasibility studies

## Abstract

**Objectives:**

People with advanced cancer express the need for support to balance everyday activities to experience quality of life. The *Balance, Activity and Quality of Life Intervention* was developed to address this need using a resource- and activity-oriented approach that integrates rehabilitation into palliative care. To inform a future full-scale evaluation, the objective of this feasibility study was to test if the selected outcome measures of health-related quality of life, including physical function and fatigue, and occupational balance could capture any possible changes of the *Balance, Activity and Quality of Life Intervention* in people with advanced cancer.

**Methods:**

Repeated-measurement feasibility study without a control group (ClinicalTrials.gov NCT04772690). Twenty-two home-living adults with advanced cancer participated in the study. The intervention was delivered at the research clinic of REPHA, The Danish Knowledge Centre for Rehabilitation and Palliative Care. Data regarding health-related quality of life, including physical function and fatigue, and occupational balance were collected with the European Organisation for Research and Treatment of Cancer Quality of Life Questionnaire Core-30 and the Occupational Balance Questionnaire at baseline, after a 5-day intervention stay and at 6- and 12-week follow-up.

**Results:**

The outcome measure of health-related quality of life captured a statistically significant improvement (*p* = 0.0046) after the 5-day intervention stay, with 64% of the participants experiencing clinically relevant improvements. No other statistically significant changes were found. Missing data were minor.

**Significance of results:**

Health-related quality of life is a promising outcome measure to capture the possible changes of the *Balance, Activity and Quality of Life Intervention*. The results indicate that a resource- and activity-oriented approach may be helpful when integrating rehabilitation into palliative care.

## Introduction

People with advanced cancer have increased life expectancy due to improvements in screening and treatment (Hashim et al. 2016). Though advanced cancer is defined as being beyond curative treatment, some types of cancer can be kept under control and be regarded as chronic conditions (National Cancer Institute 2024a; 2024b). Living with advanced cancer generally impacts the quality of life, with physical function and fatigue being commonly affected aspects (Johnsen et al. 2009; Morgan et al. 2017). As the disease progresses, people with advanced cancer experience challenges in managing and engaging in everyday activities (la Cour et al. 2009; Morgan et al. 2017; Wæhrens et al. 2020). Despite these challenges, they wish and need to continue to be engaged in meaningful everyday activities (Brose et al. 2023; von Post and Wagman 2019). This aspect may be captured in the concept of “occupational balance,” defined as the subjective experience of having the right amount and variation of everyday activities (Wagman et al. 2012). People with advanced cancer, therefore, have needs that relate both to sustaining functioning as long as possible and getting relief from pain, concerns, and grief. Collectively, these complex needs call for interventions that integrate the principles of rehabilitation into palliative care, as recently highlighted by the World Health Organization (Maribo et al. 2022; World Health Organisation 2020; World Health Organisation. Regional Office for Europe 2023). Few intervention studies exist within the field, and while some have reported positive results, further research is needed to inform rehabilitation and palliative care services for people with advanced cancer (Bayly et al. 2023; Gärtner et al. 2023; Nottelmann et al. 2019; Pilegaard et al. 2018; World Health Organisation. Regional Office for Europe 2023).

Recent studies emphasize that people with advanced cancer want to prioritize positive experiences of enjoyment and meaningfulness in the time they have left (Raunkiær 2024; von Post and Wagman 2019). In line with this, they prefer support from health professionals that focuses on resources instead of decline and problems (Johnsen et al. 2017; la Cour et al. 2020; Raunkiær 2024). This resonates with a salutogenic perspective, which focuses on factors contributing to health (Joensen et al. 2023). Hence, a resource-oriented approach should be applied when developing rehabilitation and palliative care interventions for people with advanced cancer.

The *Balance, Activity and Quality of Life Intervention* was therefore developed to support people with advanced cancer through a resource- and activity-oriented approach that integrates rehabilitation into palliative care to (1) improve health-related quality of life, including dimensions of physical function and fatigue, and (2) manage and engage in everyday activities to improve occupational balance (Pilegaard et al. 2022).

A future full-scale evaluation of the newly developed intervention requires selecting outcome measures that can capture the possible changes of the intervention (Skivington et al. 2021). Thus, it is necessary to feasibility test the preliminarily selected outcome measures, including assessing the completion rates. The feasibility study may also contribute with important knowledge to adjust the intervention content and develop the program theory of how the intervention works (O’Cathain et al. 2015; Skivington et al. 2021).

The present study aims to feasibility test if the selected outcome measures of health-related quality of life, including physical function and fatigue, and occupational balance can capture any possible changes of the *Balance, Activity and Quality of Life Intervention* in people with advanced cancer.

## Methods

### Trial design

The feasibility study was conducted as a repeated-measurement study without a control group. The feasibility study was designed to allow exploration of uncertainties in need of clarification as preparation for conducting future pilot and evaluation trials (O’Cathain et al. 2015). The study was conducted in agreement with the Helsinki Declaration (Williams 2008). The Region of Southern Denmark Data Agency approved the study (R. no. 21/13073 and R. no. 18/27843), and the study was registered at ClinicalTrials.gov (NCT04772690). Due to the nature of the study, approval from a scientific-ethical committee was not required.

### Setting

The intervention was delivered at the research clinic of REHPA, the Danish Knowledge Centre for Rehabilitation and Palliative Care in May–June 2021 and again in October–November 2021. REPHA is part of Odense University Hospital, Denmark, and offers intervention stays for people with a life-threatening illness (Rasmussen et al. 2020).

### Participants

Inclusion was based on the following criteria:

- Adult (≥18 years) living in their own home.
- Advanced or chronic cancer.
- The experience of a need for support to manage everyday activities and improve the balance between necessary activities and activities that enable enjoyment and meaningfulness.
- Ability to participate in the intervention, complete questionnaires and participate in interviews.
- Independence concerning personal care, dressing, and eating.
- Ability to speak and understand Danish.

The term “chronic” was included in the inclusion criteria and subsequent material, because the co-production process involving a panel of the target group showed that chronic cancer better represented the stage of their disease. This was also confirmed by an oncologist employed at REHPA.

Participants were recruited through: (1) specialized palliative teams at hospitals, (2) general practitioners, (3) patient associations, (4) cancer counselling services, and (5) REPHA’s website and social media. Potential participants must be assessed first by a general practitioner or oncologist at the hospitals. This health-care professional would then refer potential participants to a responsible clinical healthcare worker at REPHA, who, in collaboration with the research group, decided if the inclusion criteria were met. If in doubt, an oncologist in the department was consulted. Oral and written informed consent was obtained from all participants.

### Sample size

No requirements exist as to the number of participants needed in feasibility studies (Billingham et al. 2013). It was deemed that 20–30 persons were sufficient to feasibility test the selected outcome measures.

### Intervention

The *Balance, Activity and Quality of Life Intervention* aims to improve health-related quality of life, including physical function and fatigue, and occupational balance through a resource- and activity-oriented approach that supports positive experiences. According to the British Medical Research Council framework, the intervention was developed in a co-production process involving people with advanced cancer, professionals from REPHA and two professionals with expertise in community-based palliative rehabilitation and creative activities for people with advanced illnesses respectively. As part of the development, an intervention manual was produced together with professionals from REPHA (Pilegaard et al. 2022; Skivington et al. 2021). The intervention content was selected to maintain function and bring relief and diversion from suffering and distress. Thereby, the intervention integrated the principles of rehabilitation into palliative care to meet the needs of people with advanced cancer. The intervention consisted of 15 group-based sessions, ranging from 45 to 150 minutes, and 4 individual elements delivered by a multidisciplinary team consisting of an occupational therapist, a nurse, a social worker, and a physiotherapist, among others (Pilegaard et al. 2022). The intervention was delivered during a 5-day intervention stay and a 2-day follow-up intervention stay 6 weeks later. See Table 1 for the intervention content, and the protocol for more details (Pilegaard et al. 2022).

**Table 1.** Intervention content

Session number	Session name	Content
1	Introduction to activity, balance, and everyday life.	Introduction to the concept of occupational balance and how everyday activities affect everyday life, health, and well-being.
2	Introduction to “Walk to get happy” – activities in nature.	Introduction to how walking outside can have positive effects on physical and mental health, and discussion of how to prioritize and integrate such activity in everyday life.
3	My everyday routine and activities – introduction to diaries.	Introduction to the time-geography diary method as a useful way of examining one’s everyday activity patterns, followed by a discussion of 1 day of the diary filled out before the stay.
4	Balancing resources, fatigue, and energy – how to?	Learning about fatigue (breaks, activity adaptation, and positioning), how to plan and prioritize activities that enable meaningfulness and enjoyment, and guidance in application of assistive devices.
5	My everyday life – balance, challenges, and enjoyment.	Reflection on current occupational balance and learning how to improve balance through changes in activity patterns.
6	“Walk to get happy” – activities in nature.	Experience with physical activities in nature, both walking and different movement games.
7	Life in movement.	Reflecting on what contributes to a meaningful life, identification of important and meaningful aspects, and brainstorming how these could be a larger part of everyday life.
8	Yoga.	Experience of how breathing and relaxation exercises can give stress relief, both physically and mentally.
9	Meaningful activities: what makes you happy?	Learning about the meaning of everyday activities through phases of life, discussion of how meaningful activities have changed, for instance because of illness, and identification of activities one wants as part of one’s future life.
10	Individual conversations.	Content decided by the participant.
11	Creative expression.	Experience how creative activity can bring a state of flow, alleviate suffering, and divert attention from illness and problems. Mindfulness exercise followed by individual work on collages of important aspects of everyday life.
12	“Be good to yourself” – individual relaxing massage	Experience how massage can provide rest, well-being, and more energy.
13	Values and action plan.	Discussion of values and goals for everyday life. Prepare action plan for how the new strategies and meaningful activities can be implemented at home.
14	Midway contact: individual phone call.	Support the participants in working with their action plan.
15	Developments since last time	Discussions on how the participants have worked with their action plan and made changes to their everyday life.
16	Life in movement – family, friends, and network.	Introduction to the importance of social relationships and how they can change due to life-threatening illness. Identification of important people in the participants’ network followed by discussion.
17a	Body and movement.	Physical activity indoors and outdoors to experience enjoyment.
17b	Intimacy and sexuality.	Information about how intimacy and sexuality can be affected when living with advanced cancer.
18	Individual conversations.	Content decided by the participant.

### Data collection

Data were collected between April and December 2021. Sociodemographic data and outcome data concerning health-related quality of life, physical function, fatigue, and occupational balance were collected using questionnaires. The data were stored in REPHA’s research database. The questionnaires were sent out electronically at baseline (T1), end of the 5-day intervention stay (T2) and after 6 (T3) and 12 weeks (T4). Participants who were unable to answer the questionnaires electronically had the option of using a paper form. Two written reminders were sent 3 and 6 days after the deadline. If responses were still missing, participants were reminded by phone.

### Outcome measures

#### The European Organisation for Research and Treatment of Cancer Quality of Life Questionnaire Core-30 (EORTC QLQ-C30)

The EORTC QLQ-C30 is a cancer-specific questionnaire consisting of 30 questions addressing function, symptoms, and health-related quality of life. Items 29 and 30 measure health-related quality of life using an ordinal scale ranging from 1 to 7. The ordinal data are transformed into a score ranging from 0 to 100, where higher scores equal higher health-related quality of life. Items 1–5 constitute a sub-scale measuring self-reported physical function. Answers are scored on an ordinal scale ranging from 1 to 4 (1 = not at all, 4 = very much). The ordinal data are transformed into a score ranging from 0 to 100, where higher scores equal higher level of functioning. Items 10, 12, and 18 comprise a sub-scale measuring self-reported fatigue. Answers are scored on an ordinal scale that ranges from 1 to 4 (1 = not at all, 4 = very much). The ordinal data are transformed into a score ranging from 0 to 100, where higher scores indicate symptoms that are experienced more intensely (Fayers et al. 2001). The EORTC QLQ-C30 is assessed to be valid, reliable, and associated with high response rates (Groenvold et al. 1997).

#### The Occupational Balance Questionnaire

The Occupational Balance Questionnaire is a generic questionnaire that comprises a total of 11 items and provides an overall assessment of occupational balance based on the previous month. Each question is scored on an ordinal scale ranging from 1 to 4 (1 = completely disagree, 4 = completely agree). A sum score ranging from 11 to 44 is calculated. Higher scores indicate better occupational balance (Wagman and Håkansson 2014). The Occupational Balance Questionnaire has been found to be valid and reliable (Håkansson et al. 2020).

### Analyses

Baseline characteristics were described according to demography, educational level, job situation, and primary tumor site. Continuous, non-normally distributed data and ordinal data were described using medians and quartiles. Categorical and dichotomous data were described using numbers and percentages. Missing data were described through numbers and percentages. Changes in outcome scores were presented using box plots. Wilcoxon signed-rank test was used to test if changes in health-related quality of life, physical function, fatigue, and occupational balance from baseline to T2, T3, and T4 were statistically significant (*p* = 0.05). To determine how many participants reached a clinically relevant change of 5 points or more, a responder analysis was conducted for health-related quality of life, physical function, and fatigue (Fayers et al. 2001). Occupational balance was not included in the responder analysis as a cut-off value for clinically relevant change has not been established. Analyses were performed using STATA 17.

## Results

### Participants

**Figure 1.**
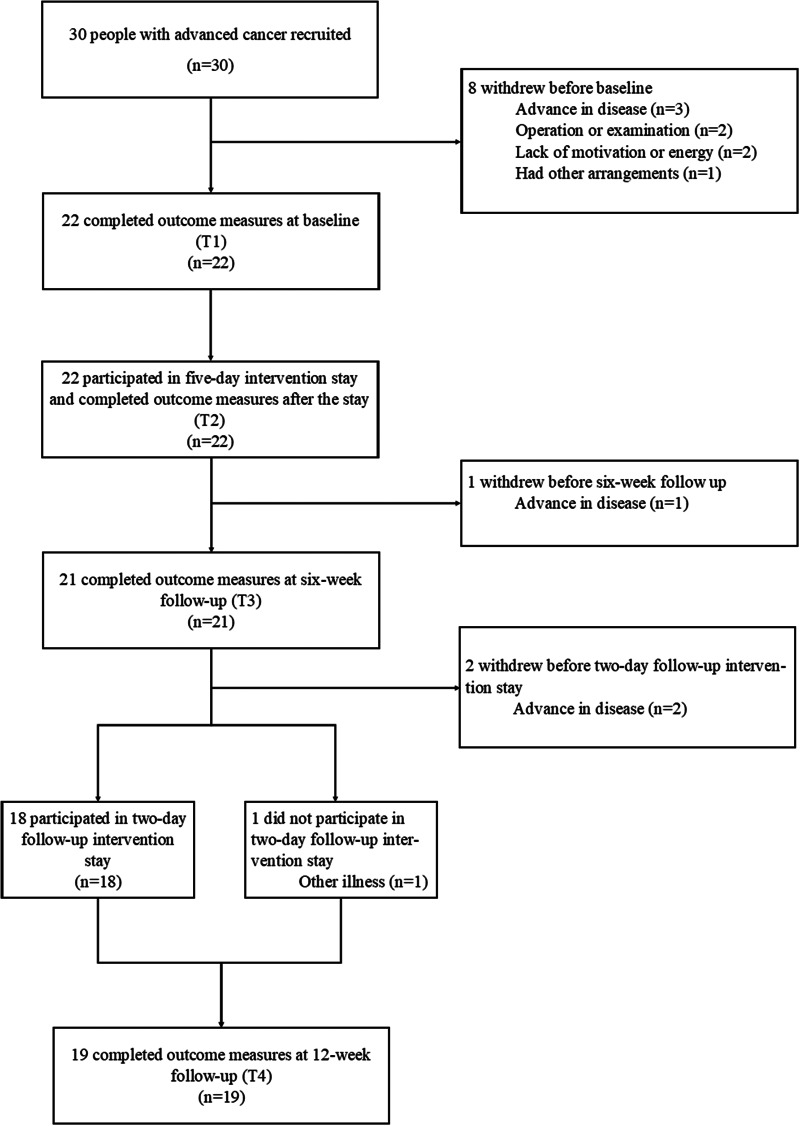
Flowchart of study participation.

Of the 30 persons initially recruited, 8 withdrew before baseline resulting in total 22 included participants (Figure 1). As shown in Figure 1, 18 participants participated in the full intervention. Of the 4 participants who did not, 1 dropped out during the 6-week follow-up, 2 during the 12-week follow-up, and the 4th did not participate in the 2-day intervention stay, but only completed the outcome measures, resulting in a total of 19 participants completing all outcome measures. The 19th participant did not withdraw from the study, but other illness hindered participation in the 2-day intervention stay, and the participant was therefore regarded as having received a smaller dose of the intervention and included in the analysis.

The participants’ baseline characteristics are presented in Table 2. The median age was 58 years, and participants were predominantly female who were living with someone. A few had children below 18 years of age, and they were generally well-educated with only 3 participants working. Cancer in breast and digestive organs were the most frequent cancer types (27%).

**Table 2.** Participants’ baseline characteristics (*N* = 22)

Age (years), median (IQR)	58 (54−68)
Women, *n* (%)	14 (64)
Living alone, *n* (%)	6 (27)
Children under 18, *n* (%)	4 (18)
Educational level, n (%)^a^	
None or semi-skilled/shorter courses	3 (14)
Vocational/skilled worker	5 (32)
Short theoretical (<3 years)	2 (9)
Long theoretical or academic (>3 years)	10 (45)
Other	1 (5)
Work status, *n* (%)^a^	
Retired	9 (41)
Full-time sick leave	7 (32)
Working part-time	3 (14)
Not working	2 (9)
Primary tumor site, *n* (%)	
Breast	6 (27)
Digestive organs	6 (27)
Prostate	2 (9)
Lung	2 (9)
Bone	1 (5)
Kidney	1 (5)
Brain	1 (5)
Skin	1 (5)
Uterus	1 (5)
Other	1 (5)

IQR = interquartile range.

a Missing: *n* = 1.

### Completion of outcome measures

Missing data not due to drop-out were minor (Table 3). Item responses were 100%, except for 2 items of the Occupational Balance Questionnaire at baseline (T1) and 1 item of the Occupational Balance Questionnaire after the 5-day intervention stay (T2).

**Table 3.** Participants completing outcome measures

Outcome measure	T1 (*N* = 22)	T2 (*N* = 22)	T3 (*N* = 21)	T4 (*N* = 19)^a^
EORTC QLQ-C30 health-related quality of life subscale, *n* (%)	22 (100)	22 (100)	21 (100)	19 (100)
EORTC QLQ-C30 physical function subscale, *n* (%)	22 (100)	22 (100)	21 (100)	19 (100)
EORTC QLQ-C30 fatigue subscale, *n* (%)	22 (100)	22 (100)	21 (100)	19 (100)
OBQ, *n* (%)				
Questions 1–5	22 (100)	22 (100)	21 (100)	19 (100)
Questions 6–7	21 (95)	22 (100)	21 (100)	19 (100)
Questions 8–10	22 (100)	22 (100)	21 (100)	19 (100)
Question 11	22 (100)	21 (95)	21 (100)	19 (100)

EORTC QLQ-C30 = European Organisation for Research and Treatment of Cancer Quality of Life Questionnaire Core-30; OBQ = Occupational Balance Questionnaire.

T1 = baseline, T2 = after the 5-day intervention stay, T3 = 6-week follow-up, T4 = 12-week follow-up.

a 19 participants answered at T4 even though 18 participated in the 2-day intervention stay.

### Changes in outcomes

**Figure 2.**
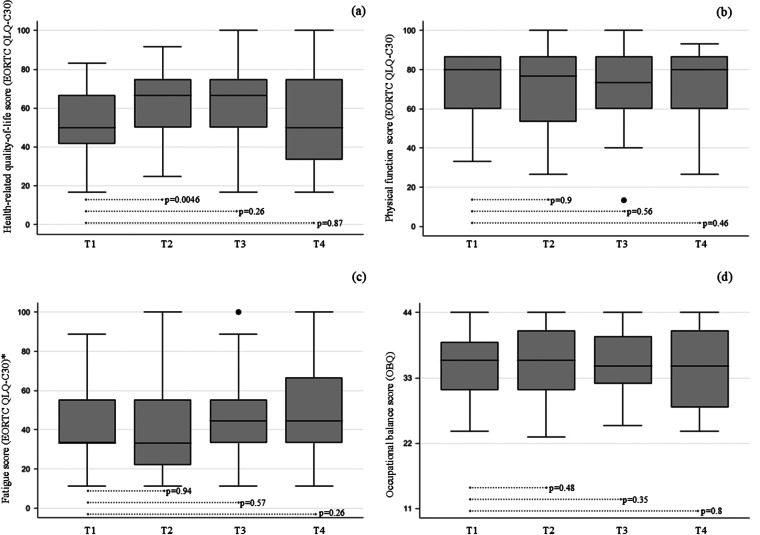
Outcome scores at each time point with p-values of change from baseline to each follow-up (Wilcoxon signed-rank test).

As presented in Figure 2, health-related quality of life was the outcome that changed the most during follow-up. From baseline to the end of the 5-day intervention stay, improvement in the median score was statistically significant (*p* = 0.0046). Improved scores were also evident at 6-week follow-up but were no longer statistically significant compared with the baseline scores. The median score dropped to baseline level at the 12-week follow-up. The median scores of physical function, fatigue, and occupational balance were almost constant at each time point, and changes compared with the baseline scores were not significant.

T1 = baseline, T2 = after the 5-day intervention stay, T3 = 6-week follow-up, T4 = 12-week follow-up.EORTC QLQ-C30 = The European Organisation for Research and Treatment of Cancer Quality of Life Questionnaire Core-30; OBQ = The Occupational Balance Questionnaire.*Increase in fatigue score represents worse symptomology.

### Responder analysis

Fourteen participants (64%) had achieved clinically relevant improvements in health-related quality of life scores from baseline to end of the 5-day intervention stay (Table 4). At 6-week follow-up, fewer participants had been able to retain the positive changes; and at 12-week follow-up, the majority reported changes for the worse. Clinically relevant positive changes regarding physical function were achieved by 10 participants (45%) at the end of the 5-day intervention stay. As with the health-related quality of life measure, these positive changes declined over time. Most of the participants had unchanged scores regarding fatigue at the end of the 5-day intervention stay and at the 6-week follow-up, but at the 12-week follow-up, many showed clinically worse scores.

**Table 4.** Participants reaching a clinically relevant change from baseline (T1)

Outcomes	End of 5-day intervention stay (T2) (*N* = 22)	6-week follow-up (T3) (*N* = 21)	12-week follow-up (T4) (*N* = 19)
Health-related quality of life, *n* (%)			
Better	14 (64)	9 (43)	7 (37)
Unchanged	4 (18)	5 (24)	2 (11)
Worse	4 (18)	7 (33)	10 (53)
Physical function, *n* (%)			
Better	10 (45)	8 (38)	7 (37)
Unchanged	3 (14)	3 (14)	4 (21)
Worse	9 (41)	10 (48)	8 (42)
Fatigue, *n* (%)			
Better	6 (27)	5 (24)	4 (21)
Unchanged	9 (41)	9 (43)	6 (32)
Worse	7 (32)	7 (33)	9 (47)

## Discussion

### Major findings

The aim of this study was to feasibility test if the selected outcome measures of health-related quality of life, including physical function and fatigue, and occupational balance could capture any possible changes of the *Balance, Activity and Quality of Life Intervention* in people with advanced cancer. The outcome measure of health-related quality of life captured a statistically significant improvement in the median score after the 5-day intervention stay, with 64% of the participants reporting a clinically relevant improvement. Scores of physical function, fatigue, and occupational balance were relatively stable during the study and showed no statistically significant changes. All outcome measures were completed with minor missing data.

### Significance of findings

In this feasibility study the outcome measure of health-related quality of life captured a statistically significant improvement in the median score, with 64% of participants reporting a clinically relevant improvement after the 5-day intervention stay of the *Balance, Activity and Quality of Life Intervention.* These results support the selection of health-related quality of life as a primary outcome measure to evaluate the intervention. The results are promising as they may indicate that the intervention was well composed to target this outcome. The intervention was targeted to improve health-related quality of life by enabling participants to engage in activities that could bring about positive experiences, such as walks in nature, creative activity, and social activities. Improvements in health-related quality of life were not evident at the 6- and 12-week follow-up. Possible explanations may include the counteraction of improvements by the progression of the disease. Alternatively, the 5-day intervention stay may have acted more as a retreat, bringing temporary relief from distress, rather than instigating sustained engagement in activities that improved health-related quality of life when the participants returned home. The findings of a recent study by a taskforce under the European Association for Palliative Care indicate that an activity-oriented approach such as that adopted in the present intervention is suitable for improving quality of life. The taskforce investigated what intervention components are considered to be effective by international researchers and health professionals when supporting people with palliative care needs. One of the intervention components identified in the study was the promotion of engagement in meaningful and/or purposeful everyday activities to improve quality of life (Wæhrens et al. 2023).

We explored possible changes in physical function and fatigue as dimensions of health-related quality of life and found that the scores regarding these outcomes were relatively stable during the study. The responder analysis showed that approximately half of the participants reported clinically relevant improvements in physical function after the 5-day intervention stay. This could indicate that these participants had positive experiences tapping into their resources through physical activities such as walks in nature and yoga. The other half of the participants reported worse physical function after the 5-day intervention stay, which suggests that they may have experienced the intervention as too strenuous or exhausting. Widespread scores indicate that the participants differed in physical function at baseline. This reflects a study of an intervention with a similar structure by Raunkiær, which found that it was important to adapt physical activity to the individual’s circumstances to avoid negative experiences (Raunkiær 2024). The experience of being confronted with declining function has been described as frustrating, confusing, painful and for some even terrifying, a reminder of illness and deterioration of the body, and as influencing one’s sense of self. (Morgan et al. 2017; Raunkiær 2024). Although such experiences have been described as having a role in the process of adapting to new ways of being engaged in everyday life, a relatively short intervention stay is possibly not an appropriate setting as the adaptation process has been found to take time and be connected to the settings, both physical and social, in which everyday life takes place (Morgan et al. 2017). Adapting activities in the intervention to avoid negative experiences of declining function is also important, considering the intervention’s resource-oriented approach to focus on positive experiences and that which is possible.

Baseline fatigue scores indicated that fatigue was not a main issue for many participants, nor were experiences of fatigue an inclusion criterion. As such, it is reasonable that the outcome measure of fatigue did not capture substantial changes during the study (Giesinger et al. 2016).

Improving health-related quality of life in people with advanced cancer has been a challenge in previous intervention studies, but studies, such as those by Temel et al. and Nottelmann et al., have demonstrated that achieving improvements in health-related quality of life is indeed possible (Johnsen et al. 2020; Nordly et al. 2019; Nottelmann et al. 2021; Pilegaard et al. 2018; Temel et al. 2010). The improved scores in health-related quality of life that we found in the present study may be related to the social aspect of being together with peers. The participants in a study evaluating a palliative rehabilitation intervention for people with advanced cancer in an outpatient setting found that it was beneficial to spend time with people who shared their situation (Nottelmann et al. 2019).

We found no improvements in occupational balance using the Occupational Balance Questionnaire. Although it was an inclusion criterion that participants had to report problems with balance in everyday activities, the baseline median score was 36 out of a maximum of 44 points, showing that the participants generally rated their occupational balance to be high (Wagman and Håkansson 2014). Because the Occupational Balance Questionnaire does not have an established cut off for imbalance, scores were not a part of the inclusion assessment. Furthermore, it is possible that the Occupational Balance Questionnaire, based on an average assessment of the previous month, is not sensitive enough to measure occupational balance in people with advanced cancer (Wagman and Håkansson 2014). Occupational balance has been explored by other researchers using a qualitative methodology. Such an approach may also be useful for investigating further if occupational balance is targeted by the present intervention (Nissmark and Malmgren Fänge 2020).

### Strengths and limitations

A strength of this study was the low drop-out rate after baseline and the high response rates, which indicate that the design successfully considered the frailty of the study population. Another strength was that the intervention was well described and based on a manual co-produced with the professionals who delivered the intervention, potentially minimizing uncertainty and misunderstandings about intervention content and delivery (O’Cathain et al. 2019; Skivington et al. 2021). In the present study, we explored uncertainties regarding selection of the most appropriate outcome measures, and a feasibility study was therefore relevant to conduct (O’Cathain et al. 2015). The design had, however, some limitations. As no control group was included in this feasibility study, we cannot be sure whether the observed changes related to the intervention or other factors. Nonetheless, our results still indicate possible changes and can thus be used to select outcome measures and further develop the intervention. The intervention was delivered at the research clinic of REHPA, which required the participants to travel to the location and be away from home for several days. These factors could have caused selection bias, as potential participants who were, for example, too frail to travel, experiencing financial difficulties since transportation was self-payed, or could not be away from children living at home were not included. This may have affected the generalizability of the results.

## Conclusion

The present study feasibility tested the outcome measures selected to evaluate the *Balance, Activity and Quality of life Intervention.* The outcome measure of health-related quality of life captured a statistically significant improvement in the median score after the 5-day intervention stay, with 64% of the participants experiencing a clinically relevant improvement. All outcome measures were completed with minor missing data. The results demonstrate that health-related quality of life is a promising primary outcome measure to capture the possible changes of the intervention in people with advanced cancer and may indicate that the intervention content succeeds in targeting this outcome. The findings can therefore inform the continued efforts to evaluate this resource- and activity-oriented intervention that integrates rehabilitation into palliative care in a municipal setting. The promising findings also indicate that a resource- and activity-oriented approach may be helpful when integrating rehabilitation into palliative care. Finally, the findings may inspire other research and clinical practice endeavoring to support people with advanced cancer.

## Data Availability

Data are saved on a secure server at the University of Southern Denmark.

## References

[ref1] Bayly J, Ahmedzai HH, Blandini MG, et al. (2023) Integrated Short-term Palliative Rehabilitation to improve quality of life and equitable care access in incurable cancer (INSPIRE): A multinational European research project. *Palliative Care and Social Practice* 17, 26323524231179979. doi:10.1177/26323524231179979 PMC10291227 37377743

[ref2] Billingham SAM, Whitehead AL and Julious SA (2013) An audit of sample sizes for pilot and feasibility trials being undertaken in the United Kingdom registered in the United Kingdom Clinical Research Network database. *BMC Medical Research Methodology* 13(1), 104–104. doi:10.1186/1471-2288-13-104 23961782 PMC3765378

[ref3] Brose JM, Willis E and Morgan DD (2023) The intentional pursuit of everyday life while dying: A longitudinal qualitative study of working-aged adults living with advanced cancer. *Palliative Medicine* 37(8), 1210–1221. doi:10.1177/02692163231180911 37310026 PMC10503259

[ref4] Fayers PM, Aaronson NK, Bjordal K, et al. (2001) *The EORTC QLQ-C30 Scoring Manual*, 3rd edn. Brussels: European Organisation for Research and Treatment of Cancer.

[ref5] Gärtner HS, Shabnam J, Aagesen M, et al. (2023) Combined rehabilitation and palliative care interventions for patients with life-threatening diseases – PREGOAL. A scoping review of intervention programme goals. *Disability & Rehabilitation*, 1–10. doi:10.1080/09638288.2023.2246373 37580981

[ref6] Giesinger JM, Kuijpers W, Young T, et al. (2016) Thresholds for clinical importance for four key domains of the EORTC QLQ-C30: Physical functioning, emotional functioning, fatigue and pain. *Health and Quality of Life Outcomes* 14(1), 87. doi:10.1186/s12955-016-0489-4 27267486 PMC4897949

[ref7] Groenvold M, Klee MC, Sprangers MAG, et al. (1997) Validation of the EORTC QLQ-C30 quality of life questionnaire through combined qualitative and quantitative assessment of patient-observer agreement. *Journal of Clinical Epidemiology* 50(4), 441–450. doi:10.1016/S0895-4356(96)00428-3 9179103

[ref8] Håkansson C, Wagman P and Hagell P (2020) Construct validity of a revised version of the Occupational Balance Questionnaire. *Scandinavian Journal of Occupational Therapy* 27(6), 441–449. doi:10.1080/11038128.2019.1660801 31524026

[ref9] Hashim D, Boffetta P, La Vecchia C, et al. (2016) The global decrease in cancer mortality: Trends and disparities. *Annals of Oncology* 27(5), 926–933. doi:10.1093/annonc/mdw027 26802157

[ref10] Joensen MB, Lindahl-Jacobsen L, Lindahl M, et al. (2023) Making meaning of everyday life in the context of lung cancer treatment—A qualitative study of outpatients’ perspectives. *Scandinavian Journal of Occupational Therapy*, 1–11. doi:10.1080/11038128.2023.2249043 37625436

[ref11] Johnsen AT, Eskildsen NB, Thomsen TG, et al. (2017) Conceptualizing patient empowerment in cancer follow-up by combining theory and qualitative data. *Acta Oncologica* 56(2), 232–238. doi:10.1080/0284186x.2016.1267403 28080181

[ref12] Johnsen AT, Petersen MA, Pedersen L, et al. (2009) Symptoms and problems in a nationally representative sample of advanced cancer patients. *Palliative Medicine* 23(6), 491–501. doi:10.1177/0269216309105400 19443525

[ref13] Johnsen AT, Petersen MA, Sjøgren P, et al. (2020) Exploratory analyses of the Danish Palliative Care Trial (DanPaCT): A randomized trial of early specialized palliative care plus standard care versus standard care in advanced cancer patients. *Supportive Care in Cancer* 28(5), 2145–2155. doi:10.1007/s00520-019-05021-7 31410598

[ref14] la Cour K, Gregersen Oestergaard L, Brandt Å, et al. (2020) Process evaluation of the Cancer Home-Life Intervention: What can we learn from it for future intervention studies? *Palliative Medicine* 34(10), 1425–1435. doi:10.1177/0269216320939227 32611224

[ref15] la Cour K, Nordell K and Josephsson S (2009) Everyday lives of people with advanced cancer: Activity, time, location, and experience: Occupation, participation and health. *OTJR* 29(4), 154–162. doi:10.3928/15394492-20090914-03

[ref16] Maribo T, Ibsen C, Thuesen J, et al. (eds) (2022) *Hvidbog Om Rehabilitering [White Book about Rehabilitation]*. Aarhus: Rehabiliteringsforum Danmark.

[ref17] Morgan DD, Currow DC, Denehy L, et al. (2017) Living actively in the face of impending death: Constantly adjusting to bodily decline at the end-of-life. *BMJ Supportive & Palliative Care* 7(2), 179–188. doi:10.1136/bmjspcare-2014-000744 26182946

[ref18] National Cancer Institute (2024a) Advanced cancer. https://www.cancer.gov/publications/dictionaries/cancer-terms/def/advanced-cancer (accessed 30 April 2024).

[ref19] National Cancer Institute (2024b) Chronic disease. https://www.cancer.gov/publications/dictionaries/cancer-terms/def/chronic-disease (accessed 30 April 2024).

[ref20] Nissmark S and Malmgren Fänge A (2020) Occupational balance among family members of people in palliative care. *Scandinavian Journal of Occupational Therapy* 27(7), 500–506. doi:10.1080/11038128.2018.1483421 30001672

[ref21] Nordly M, Benthien KS, Vadstrup ES, et al. (2019) Systematic fast-track transition from oncological treatment to dyadic specialized palliative home care: DOMUS – A randomized clinical trial. *Palliative Medicine* 33(2), 135–149. doi:10.1177/0269216318811269 30415608

[ref22] Nottelmann L, Groenvold M, Vejlgaard TB, et al. (2021) Early, integrated palliative rehabilitation improves quality of life of patients with newly diagnosed advanced cancer: The Pal-Rehab randomized controlled trial. *Palliative Medicine* 35(7), 1344–1355. doi:10.1177/02692163211015574 34000886

[ref23] Nottelmann L, Jensen LH, Vejlgaard TB, et al. (2019) A new model of early, integrated palliative care: Palliative rehabilitation for newly diagnosed patients with non-resectable cancer. *Supportive Care in Cancer* 27(9), 3291–3300. doi:10.1007/s00520-018-4629-8 30612238

[ref24] O’Cathain A, Croot L, Duncan E, et al. (2019) Guidance on how to develop complex interventions to improve health and healthcare. *BMJ Open* 9(8), e029954. doi:10.1136/bmjopen-2019-029954 PMC6701588 31420394

[ref25] O’Cathain A, Hoddinott P, Lewin S, et al. (2015) Maximising the impact of qualitative research in feasibility studies for randomised controlled trials: Guidance for researchers. *Pilot and Feasibility Studies* 1(32). doi:10.1186/s40814-015-0026-y PMC5154038 27965810

[ref26] Pilegaard MS, la Cour K, Gregersen Oestergaard L, et al. (2018) The ‘Cancer Home-Life Intervention’: A randomised controlled trial evaluating the efficacy of an occupational therapy–based intervention in people with advanced cancer. *Palliative Medicine* 32(4), 744–756. doi:10.1177/0269216317747199 29299957 PMC5881790

[ref27] Pilegaard MS, Timm H, Birkemose HK, et al. (2022) A resource-oriented intervention addressing balance in everyday activities and quality of life in people with advanced cancer: Protocol for a feasibility study. *Pilot and Feasibility Studies* 8(1), 86. doi:10.1186/s40814-022-01038-8 35443699 PMC9019951

[ref28] Rasmussen A, Jespersen E, Backmann T, et al. (eds) (2020) *Praksisbeskrivelser – forskningsklinik REHPA. Standard Rehabiliteringsforløb for Mennesker Med Eller Efter Kræft*. REHPA, Videncenter for Rehabilitering og Palliation. https://www.rehpa.dk/wp-content/uploads/2020/04/Rehpa_praksisbeskrivelser_020420_enkelt.final_.pdf.

[ref29] Raunkiær M (2024) The experiences of people with advanced cancer and professionals participating in a program with focus on rehabilitation and palliative care. *OMEGA - Journal of Death and Dying* 88(4), 1383–1402. doi:10.1177/00302228211058307 35000465

[ref30] Skivington K, Matthews L, Simpson SA, et al. (2021) A new framework for developing and evaluating complex interventions: Update of Medical Research Council guidance. *BMJ (Online)* 374, 2061. doi:10.1136/bmj.n2061 PMC8482308 34593508

[ref31] Temel JS, Greer JA, Muzikansky A, et al. (2010) Early palliative care for patients with metastatic non–small-cell lung cancer. *New England Journal of Medicine* 363(8), 733–742. doi:10.1056/NEJMoa1000678 20818875

[ref32] von Post H and Wagman P (2019) What is important to patients in palliative care? A scoping review of the patient’s perspective. *Scandinavian Journal of Occupational Therapy* 26(1), 1–8. doi:10.1080/11038128.2017.1378715 28937317

[ref33] Wæhrens EE, Brandt Å, Peoples H, et al. (2020) Everyday activities when living at home with advanced cancer: A cross-sectional study. *European Journal of Cancer Care (Engl)* 29(5), e13258. doi:10.1111/ecc.13258 32489002

[ref34] Wæhrens EE, Morgan DD, la Cour K, et al. (2023) International consensus on occupational therapy interventions for people with palliative care needs: A European Association for Palliative Care Group Concept Mapping study. *Palliative Medicine*, 2692163231188155–2692163231188155. doi:10.1177/02692163231188155 37534430

[ref35] Wagman P and Håkansson C (2014) Introducing the Occupational Balance Questionnaire (OBQ). *Scandinavian Journal of Occupational Therapy* 21(3), 227–231. doi:10.3109/11038128.2014.900571 24649971

[ref36] Wagman P, Håkansson C and Björklund A (2012) Occupational balance as used in occupational therapy: A concept analysis. *Scandinavian Journal of Occupational Therapy* 19(4), 322–327. doi:10.3109/11038128.2011.596219 21780985

[ref37] Williams JR (2008) The Declaration of Helsinki and public health. *Bulletin World Health Organisation* 86(8), 650–652. doi:10.2471/blt.08.050955 PMC2649471 18797627

[ref38] World Health Organisation (2020) Palliative care. https://www.who.int/news-room/fact-sheets/detail/palliative-care (accessed 30 April 2024).

[ref39] World Health Organisation. Regional Office for Europe (2023) Policy brief on integrating rehabilitation into palliative care services, 2023. Copenhagen. https://apps.who.int/iris/handle/10665/366505 (accessed 30 April 2024).

