# Promising results of a resource- and activity-oriented intervention integrating rehabilitation into palliative care in people with advanced cancer: A feasibility study testing outcome measures – CORRIGENDUM

**DOI:** 10.1017/S1478951525100643

**Published:** 2025-09-17

**Authors:** Marie Brunsgaard Laursen, Marc Sampedro Pilegaard, Karen la Cour

**Keywords:** palliative care, rehabilitation, quality of life, neoplasms, feasibility studies, corrigendum

The Authors apologise for an error in the published article.

Analysis of data related to this study found that one participant had not been registered accurately. The participant did not attend the two-day follow up stay, but did answer all outcomes measures. Accordingly, on page 4 the text should be:

As shown in Figure 1, 17 participants participated in the full intervention. Of the 5 participants who did not, 1 dropped out during the 6-week follow-up, 2 during the 12-week follow-up, and 2 did not participate in the 2-day intervention stay, but only completed the outcome measures, resulting in a total of 19 participants completing all outcome measures. These two participants did not withdraw from the study, but disease progression and other illness hindered participation in the 2-day intervention stay, and they were therefore regarded as having received a smaller dose of the intervention and included in the analysis.

instead of

As shown in Figure 1, 18 participants participated in the full intervention. Of the 4 participants who did not, 1 dropped out during the 6-week follow-up, 2 during the 12-week follow-up, and the 4th did not participate in the 2-day intervention stay, but only completed the outcome measures, resulting in a total of 19 participants completing all outcome measures. The 19th participant did not withdraw from the study, but other illness hindered participation in the 2-day intervention stay, and the participant was therefore regarded as having received a smaller dose of the intervention and included in the analysis.

The same correction applies to figure 1.
